# A modified theoretical framework to assess implementation fidelity of adaptive public health interventions

**DOI:** 10.1186/s13012-016-0457-8

**Published:** 2016-07-08

**Authors:** Dennis Pérez, Patrick Van der Stuyft, Maríadel Carmen Zabala, Marta Castro, Pierre Lefèvre

**Affiliations:** 1 Epidemiology Division, Tropical Medicine Institute “Pedro Kouri”, Autopista Novia del Mediodía, Km. 6 ½, La Lisa, Marianao 13, PO Box 601, Havana City, Cuba; 2 Department of Public Health, Institute of Tropical Medicine, Nationalestraat 155, 2000 Antwerp, Belgium; 3 Department of Public Health, Ghent University, Ghent, Belgium; 4 Latin-American Faculty of Social Sciences (FLACSO), Habana University, Havana, Cuba

**Keywords:** Implementation, Fidelity, Adaptation, Reinvention, Adaptive interventions, Conceptual framework, Translating research, Cuba

## Abstract

**Background:**

One of the major debates in implementation research turns around fidelity and adaptation. Fidelity is the degree to which an intervention is implemented as intended by its developers. It is meant to ensure that the intervention maintains its intended effects. Adaptation is the process of implementers or users bringing changes to the original design of an intervention. Depending on the nature of the modifications brought, adaptation could either be potentially positive or could carry the risk of threatening the theoretical basis of the intervention, resulting in a negative effect on expected outcomes. Adaptive interventions are those for which adaptation is allowed or even encouraged. Classical fidelity dimensions and conceptual frameworks do not address the issue of how to adapt an intervention while still maintaining its effectiveness.

**Discussion:**

We support the idea that fidelity and adaptation co-exist and that adaptations can impact either positively or negatively on the intervention’s effectiveness. For adaptive interventions, research should answer the question how an adequate fidelity-adaptation balance can be reached. One way to address this issue is by looking systematically at the aspects of an intervention that are being adapted. We conducted fidelity research on the implementation of an empowerment strategy for dengue prevention in Cuba. In view of the adaptive nature of the strategy, we anticipated that the classical fidelity dimensions would be of limited use for assessing adaptations. The typology we used in the assessment—implemented, not-implemented, modified, or added components of the strategy—also had limitations. It did not allow us to answer the question which of the modifications introduced in the strategy contributed to or distracted from outcomes. We confronted our empirical research with existing literature on fidelity, and as a result, considered that the framework for implementation fidelity proposed by Carroll et al. in 2007 could potentially meet our concerns. We propose modifications to the framework to assess both fidelity and adaptation.

**Summary:**

The modified Carroll et al.’s framework we propose may permit a comprehensive assessment of the implementation fidelity-adaptation balance required when implementing adaptive interventions, but more empirical research is needed to validate it.

## Background

Some authors argue that intervention research is a set of sequential studies (i.e., efficacy, effectiveness, and dissemination) that provides different kinds of evidence about an intervention [1, 2]. Rychectnik et al. [3], based on Nutbean and Bauman [4], formulated how to proceed while building evidence for innovative interventions: assessing process and outcome/impact of the intervention (intervention testing); determining if similar outcomes can be reproduced when the intervention is adapted to other settings or populations (intervention replication); and examining real population outcomes and public health benefits (intervention dissemination). Replication and dissemination studies are rooted on the assumption of the potential translation of evidence-based interventions to new settings and hinge on implementation issues.

Implementation is a specific set of purposeful processes and activities designed to put into practice an intervention or program of known dimensions [5], which requires to be measured with outcomes that are conceptually and empirically distinct from those to assess intervention effectiveness [5–7]. Distinguishing between “implementation” and “intervention” outcomes is critical. When translation efforts fail, it helps to determine if the failure occurred because the intervention was ineffective (intervention failure) or whether it was deployed incorrectly (implementation failure) [7].

Translating evidence-based health interventions has resulted in one of the major dilemmas in implementation research: fidelity versus adaptation [8, 9]. Fidelity or the degree to which an intervention is implemented as intended by its developers [8–14] is an implementation outcome [7] that is particularly meant to ensure that the intervention maintains its intended effects [8–14]. On the opposite, adaptation is the process of bringing changes to the original design of an intervention by its implementers or users [9, 11, 12]. Fidelity and adaptation are closely linked but remain two opposed concepts. The highest the level of the fidelity achieved, the less there are changes brought to the original design of an intervention. Inversely, the more an intervention is adapted, the more likely the fidelity can be threatened.

Several studies have demonstrated that the fidelity with which an intervention is implemented affects its effectiveness [8–15]. Hence, achieving high fidelity has been the overriding concern for many researchers who struggle to move from efficacy studies to real-world implementation, in particular in the field of pharmacological and psychosocial interventions designed to treat specific health problems [16–18]. However, in practice, the adaptation of interventions has been the rule rather than the exception [9, 10]. Moreover, some authors have argued that certain interventions might need to be adapted in the course of its implementation [9, 11, 12]. This is the case of *adaptive interventions*. We define these as interventions for which stakeholders are allowed, or even encouraged, to bring changes to the original design. This definition includes the type of adaptive intervention as defined by Collins et al. [19] and Nahum-Shani et al. [20], where pre-defined changes are allowed by the intervention developers. For this type of adaptive interventions, fidelity is important to ensure that pre-defined adaptations occurred as intended. However, adaptive interventions also include not pre-defined changes originating from implementers. This kind of changes occur more in the context of complex public health interventions involving different organizational levels and targeting collective behaviors than in interventions targeting individuals with different needs and where the control of the implementation process by the intervention developers is not possible or even desirable.

Examples of such adaptive interventions are empowerment strategies for disease prevention and control. Empowerment is a process through which individuals, groups, and communities are provided with the capabilities to take power over decisions that affect their lives [21, 22]. Indeed, promoting participation in decision-making implies a high degree of uncertainty as to what will be planed and/or achieved. In all cases, depending on the nature of the modifications brought to the original design of an intervention, adaptation could either be potentially positive or could carry the risk of threatening the theoretical basis of the intervention, resulting in a negative effect on expected outcomes [8, 12].

While five dimensions [adherence, dose, quality of delivery, participant responsiveness, and program differentiation] have been put forward and are commonly used for measuring fidelity [8–15], there has been little research or practical advice on how to adapt an intervention to maintain its effective ingredients and mechanisms [12]. On the basis of a critical systematic review of existing conceptualizations of implementation fidelity, Carroll et al. [15] proposed a conceptual framework for understanding and measuring this concept. They acknowledged that adaptations are likely to occur in real-world implementation, but the question on how to address this issue while measuring fidelity remained unanswered.

In this debate paper, we argue that for adaptive interventions, the issue of fidelity cannot be apprehended independently from the issue of adaptation and that both concepts are intrinsically linked. We then propose a modified Carroll et al.’s framework for implementation fidelity bringing together literature on fidelity and results from our empirical research in this field in order to provide a better fit for adaptive interventions.

### Carroll et al.’s conceptual framework for implementation fidelity

As previously stated, in the last decade-and-a-half, the concept of implementation fidelity has been described and defined in terms of five dimensions that need to be measured: adherence—program implementation as described; dose—frequency and duration of the exposure to the program; quality of delivery—manner in which the program is delivered; participant responsiveness—the degree to which participants are engaged; and program differentiation—critical features that distinguishes the program [8–14]. While some authors argue that each of these dimensions is an alternative way to measure fidelity, it has been also argued that a comprehensive picture of fidelity requires the measurement of all the five dimensions [15].

Carroll et al. [15] proposed their conceptual framework in an attempt to “attribute meaning” to the concept of fidelity, but also to clarify and explain the function of each of the five classical fidelity dimensions and their relationship to one another. In their framework, they also included two additional elements suggested by a broader literature review on diffusion of innovations and on implementation fidelity: intervention complexity and facilitation strategies. These are strategies put in place to optimize the level of fidelity achieved.

**Fig. 1:**
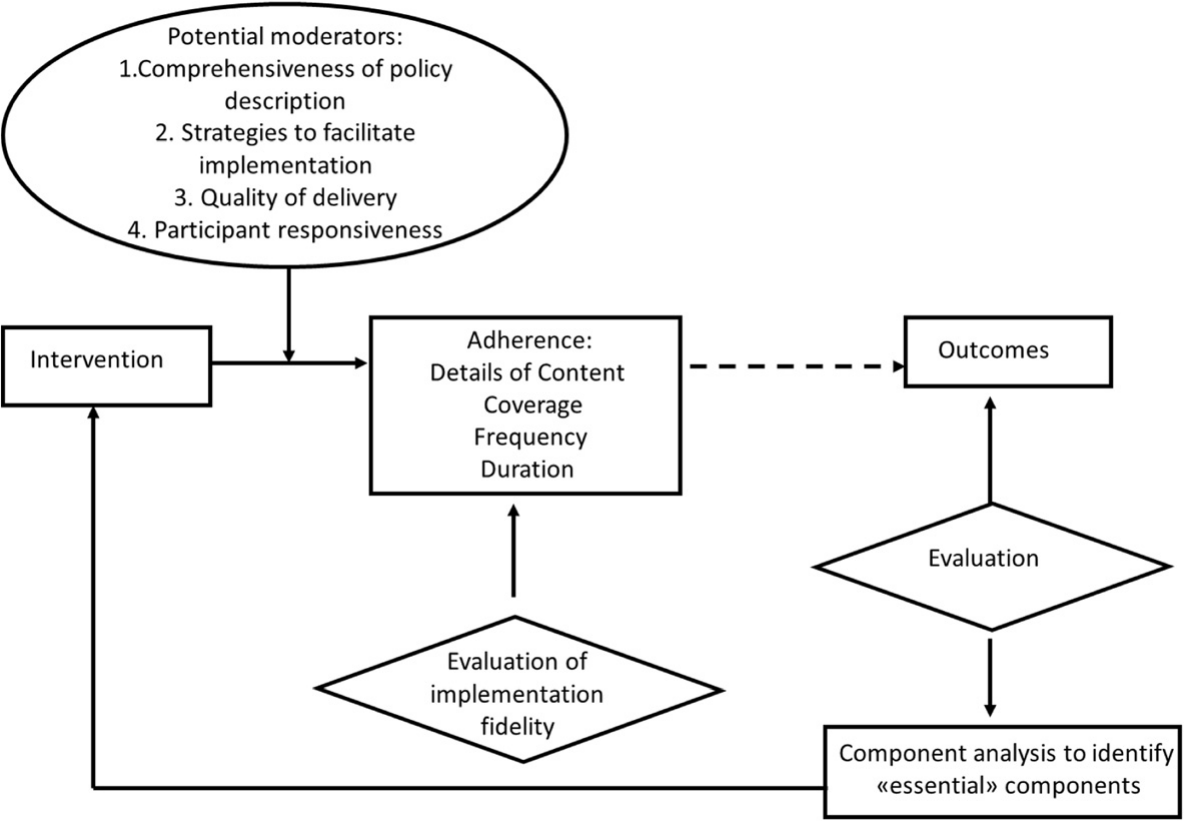
Conceptual framework for implementation fidelity proposed by Carroll et al. in 2007

For Carroll et al. [15], “the measurement of implementation fidelity is the measurement of adherence, i.e., how far those responsible for delivering an intervention actually adhere to the intervention as it is outlined by its designers. Adherence includes the subcategories of content, frequency, duration and coverage (i.e., dose). The degree to which the intended content or frequency of an intervention is implemented is the degree of implementation fidelity achieved for that intervention. The level achieved may be influenced or affected, (i.e., moderated) by certain other variables: intervention complexity, facilitation strategies, quality of delivery, and participant responsiveness” (Fig. 1). The broken line in Fig. 1 indicates that the relationship between an intervention and its outcomes is external to implementation fidelity, but that the degree of implementation fidelity achieved can affect this relationship.

According to Carroll et al. [15], in the real world, an intervention cannot be always fully implemented as planned. An intervention may also be implemented successfully, and meaningfully, if only its essential components are implemented. An analysis of outcomes may help to identify those components that are essential to the intervention, if the intervention is to maintain its intended effects. Outcome evaluation may also inform the content of the intervention by determining the minimum requirements for high implementation fidelity, i.e., the essential components.

Carroll et al. [15] stated that identifying essential components provides scope for adaptation but that the question on how to identify what is essential remains unanswered. They suggested that a possible way to identify what is essential could be conducting sensitivity or component analysis using implementation fidelity data and performance outcomes from different studies of the same intervention.

### The implementation process and the need for adaptation

Dusenbury et al. [8] conducted a literature review on research on the fidelity of implementation in different fields (e. g., mental health, prevention of psychopathology, personal and social competence promotion, education, drug abuse treatment and prevention) published over a 25-year period. In this review, the authors discussed the tensions between fidelity of implementation and the need for adaptation [8]. They concluded that research has not yet indicated whether and under what conditions adaptation might enhance program outcomes and under which conditions it results in a loss of effectiveness.

Although there is a general agreement that adaptation entails bringing changes to the original design of an intervention [9–15, 23–26], there is no common agreement on the definition of adaptation [9]. Restrictive definitions co-exist with broader understandings. In restrictive definitions, adaptations are limited to tailoring interventions to local contexts and circumstances [5, 19, 20]. In a broader sense, adaptation is the result of the cognitive processes that occur while implementers or potential users struggle to give meaning to an intervention during its implementation [23]. In this understanding of the term, originating from diffusion of innovation theory scholars, this specific type of adaptation, which goes further than simply adapting, is very often called *reinvention*. We situate ourselves in this line of thinking.

Rogers [23], in his solid and well-articulated diffusion of innovations theory, brings together central concepts and issues regarding widespread implementation (e.g., dissemination, replication, sustainability, institutionalization, routinization, and fidelity) that have been discussed in the literature at large [27–31]. His theory is built on observations of regularities and patterns in the diffusion of a wide range of innovations (i.e., idea, practice, or object that is perceived as new by its potential individual and organizational users), in different cultural contexts with different users, as well as on a theoretical reflection on the issue that extends over decades.

Diffusion of innovations theory dismantles some of the arguments of early diffusion studies supporting the idea that an innovation is an invariant, which does not change throughout the diffusion process; and that potential users are passive subjects that implement an innovation with fidelity, i.e., just as intended by its developers. In Rogers’ view [23], reinvention (i.e., a specific type of adaptation) or the degree to which an innovation is changed or modified occurs at the implementation stage for many potential users and leads to faster and sustainable adoption of the innovation.

Bauman, Stein, and Ireys [25] introduced the idea of program “uniqueness” referring to the specifics and unusual conditions under which programs are created that are not present in actual implementation. This idea brings us back to an understanding of “translating” evidence-based interventions as the art to achieve an equivalent rather than a literal copy [2, 32]. Backer [11] argued that adaptations of some features of innovative interventions are inevitable, even desirable, to maintain the theory-based outcomes. On the one hand, the inability to modify programs may produce users’ resistance. Besides, a rigid position regarding fidelity can lead to innovations that are irrelevant or even inappropriate for certain users. Backer [11] also states that interventions that are flexible and that can be adapted have a better chance to fit a wider range of users. Certain users may have an aversion to simply “copying” interventions and pressurize for recognition of *their* adaptations [11].

Furthermore, according to some authors [11, 33], program fidelity is underpinned by a professionally driven or “top-down” approach to implementation, while adaptation seems to be closer to a user-based or “bottom-up” approach, which is more politically appealing to promoters of social development. Consequently, some authors state that a certain amount of adaptation is needed in order to achieve users’ involvement and ownership for successful implementation of some innovations [34, 35]. This is particularly relevant for adaptive interventions. These require emphasizing the processes that permit them to be modified and revised according to their unfolding interaction within the institutional setting and context [36]. This connects with the idea of *mutual adaptation*, i.e., adaptation of both the intervention and of the host organization [11, 36–38], as organizational changes will—need to—occur within the institutional setting to accommodate the intervention [5].

If there is no agreement in the literature on defining adaptation, another challenge is the lack of consensus on how to operationalize these concepts [9]. Still, some typologies are found in the literature [11, 24, 39].

Adaptations can be deliberate or accidental and include (1) additions of new components; (2) deletions or radical modifications to an intervention component in such a way that it no longer resembles the original one; and (3) minor or major modifications to an existing intervention component [11, 24] (e.g., changes in the nature of program components, in the manner or intensity of administration, cultural modifications required by local circumstances [11]). According to Rebchook et al. [24], in theory, the implications of the above three kinds of adaptations on fidelity are different. When adding something new, fidelity can be easily maintained. When a component is suppressed or radically modified, fidelity is threatened. In the third case, depending upon what the modifications entail, it may or may not affect fidelity. More recently, Stirman et al. [39] proposed an elaborated framework for classifying modifications brought to evidence-based programs or interventions. It encompasses what is modified, by whom, at which level of delivery, contextual modifications and the nature of the modifications introduced in the content of the intervention.

In the literature, authors deal with adaptation in very different ways. Bellg and colleagues do not take into consideration adaptation and are exclusively concerned with documenting, monitoring, and enhancing fidelity [16–18]. For some authors such as Collins, Murphy, and Bierman [19], adaptation of intervention options (e.g., dosage) can only be decided upon by intervention developers and follows a sequence of decision rules that recommend when and how the intervention should be modified with the aim to optimize long-term effectiveness. Other authors [5, 40, 41] support the idea that interventions should first be implemented with fidelity before allowing for adaptation, in order to discriminate between desirable and undesirable changes. Finally, for Carroll et al. [15], adaptation is likely to occur, but they argue that as far as essential components are not known, fidelity to the whole intervention is required.

We support the idea that fidelity and adaptation co-exist [23]. From our point of view, adaptations can lead either to improve on or to threaten the intervention’s underlying theory of change [42] and thus impact positively or negatively on effectiveness. For adaptive interventions, research should answer the question how an adequate fidelity-adaptation balance can be reached. One way to address this issue is looking systematically at the aspects of an intervention that are being adapted [12].

### Our empirical research: fidelity-adaptation balance in the implementation of an empowerment strategy

Dengue is a vector-borne disease that is transmitted by an *Aedes* mosquito infected with one of the four dengue viruses [43, 44]. A mild episode of the disease can evolve to a severe and fatal hemorrhagic illness [45]. The disease is of growing public health importance in tropical and subtropical areas [46]. All currently available control methods target the *Aedes* mosquito, and it is nearly impossible to implement them without community acceptance or active involvement [47–50].

Empowerment strategies have been reported as effective for community-based dengue prevention and control in Cuba [51–55] and elsewhere [56–58]. Still, empowerment strategies remain controversial due to transferability and scalability issues [59], and evidence available in the literature on participatory implementation processes is scarce [60].

We conducted in the Cuban context a fidelity assessment of the implementation of an evidence-based empowerment strategy aiming at community involvement in decision-making on dengue vector control activities when it was replicated at intermediate scale [61]. The empowerment strategy was developed by researchers from the Pedro Kourí Institute of Tropical Medicine (IPK) in Havana City. It was defined in terms of components and subcomponents. The components were capacity building, organization and management, community work, and surveillance.

The strategy was implemented between October 2004 and December 2007 in 16 communities (circumscriptions) randomly selected within three People’s Councils (PCs) in La Lisa municipality of Havana City [55]. The circumscription is the lowest level of local government and covers about 1000 inhabitants. The PCs are intermediate government structures between the municipalities and the circumscriptions. Circumscriptions and PCs were heterogeneous in terms of socio-demographic composition of the population, previous experiences with participation, characteristics of the leadership, and resources and dynamic of the local government, among others.

In view of the adaptive nature of the strategy, we anticipated that the classical fidelity dimensions would be of limited use for assessing adaptations. We therefore opted to assess fidelity and adaptation in the implementation of the strategy based on Rebchoock et al.’s [24] typology to determine implemented, not-implemented, modified or added components, and subcomponents at circumscription level.

A three-step assessment was conducted [61]: (1) an individual evaluation by three strategy developers involved as facilitators in the implementation of the strategy, based on the analysis of proceedings and minutes of capacity-building workshops and process documentation forms that were filled in by implementers (i.e., implementation descriptors of component/subcomponents of the strategy for each circumscription); (2) a discussion of these assessments by a broader group of strategy developers (the three from the first step and three additional ones). If the six agreed that in a given circumscription, a component or subcomponent of the strategy was implemented as intended, it was classified as implemented. If all agreed that a component or subcomponent was not implemented, it was classified as such. If any of the professionals judged that a component or subcomponent was modified, it was classified as such. Added activities were also identified; and (3) the assessment was consensually refined following its discussion with implementers in a participatory evaluation workshop.

Qualitative data obtained from the three-step assessment were transformed into quantitative data [62]. Frequencies of not-implemented, modified, and implemented subcomponents were tabulated over all circumscriptions, and the average was calculated for the four components. To explore the relationships among the components, not-implemented, modified, and implemented components of the strategy were scored 0, 1, and 2, respectively, and their values were summed for each circumscription. In addition, semi-structured interviews were conducted with 13 implementers and deductively analyzed to identify possible explanations for the observed variation in the implementation of the strategy. The assessment was conducted retrospectively, as part of the final evaluation of the replication of the strategy. Table 1 provides a description of the empowerment strategy for dengue vector control carried out in La Lisa municipality by components and subcomponents as well as the number of circumscriptions that implemented as intended, modified, or did not implement subcomponents.

**Table 1:** The empowerment strategy for dengue vector control by components and subcomponents and the number of circumscriptions (*n* = 16) that implemented as intended, modified, or did not implement subcomponents. La Lisa municipality, Havana City, 2004–2007

Components	Operational definition of the components	Subcomponents	Implemented as intended	Modified	Did not implement
1. Capacity-building	Development of knowledge, capabilities and associated values, and practices required by community members to lead community empowerment for dengue vector control	1.1 Diagnosis, group work, and participation	10	1	5
		1.2 Surveillance of risks and behaviors	11	5	0
		1.3 Action plans and communication strategy	6	5	5
		1.4 Participatory evaluation	8	0	8
2. Organization and management	The way the stakeholders involved in dengue vector control establish themselves, set commitments and roles, identify resources, and make decisions	2.1 Presence of community working groups (CWGs) leading the strategy	7	4	5
		2.2 Vector control program staff within CWGs	4	7	5
		2.3 Community resources identified	3	1	12
		2.4 External resources mobilized	3	0	13
3. Community work	Repetitive cycle of actions developed by a group of community members to change the conditions that increase the probability of dengue transmission	3.1 Risk mapping	11	1	4
		3.2 Problem assessment	10	2	4
		3.3 Action plan	7	1	8
		3.4 Actions executed	7	0	9
		3.5 Communication strategy	5	5	6
		3.6 Elaboration of communication materials	3	3	10
		3.7 Monitoring and evaluation	2	0	14
4. Surveillance	Timely and systematically organized data collection and analysis on dengue transmission risks and associated behaviors in order to take actions	4.1 Identification of environmental risks	16	0	0
		4.2 Identification of domiciliary risks	13	0	3
		4.3 Identification of associated behaviors	6	0	10

Surveillance was the most implemented component followed by capacity building. Community work and organization and management were less implemented or modified [61]. Even in the case of the more implemented components, there were some subcomponents not implemented. The more components and subcomponents were innovative (i.e., those that implied activities which were far-off from or disruptive of the routine vector control practices and that could not be easily accommodated by implementers’ expertise, previous experiences or know-how), the less they were implemented.

Scarcely implemented subcomponents were internal and external resource mobilization by the circumscriptions, designing communication strategies, and developing local communication materials. Main modifications introduced were the composition of CWGs, changing the approach of the capacity building from participatory to individualized and modifications to the design of the training activities such as using more adequate participatory techniques adapted to the characteristics of the participants. Within the whole strategy, some activities were added such as linking the strategy with activities conducted at primary school level, involving stakeholders not initially foreseen, self-organizing additional community training workshops, and conducting strength assessments as part of the participatory community diagnosis.

The difficulties encountered during implementation were related to appropriate training and skills, available time, lack of support and commitment to the strategy by the local government and health authorities, lack of motivation of local leadership, and integration of actors and resources. The study showed a wide variability of fidelity in the implementation of the intervention. The variability was largely explained by the complexity of the strategy and the lack of knowledge on its basic principles among the implementers*.* The variation in implementation fidelity did not result in a substantial loss of effectiveness [55]. More detailed information on the methods used in our fidelity study and results are provided in Pérez et al. 2010 [61].

Rebchook et al.’s [24] typology proved suitable to assess the fidelity-adaptation balance of the empowerment strategy, but had limitations. It did not allow us to answer the question “which of the modifications introduced in the strategy contributed to, or distracted from, outcomes?” Rebchook et al.’s assertion that adding new components or subcomponents to an original design a priori does not threaten fidelity, is questionable; such components could contradict the basic principles of the intervention. Moreover, using Rebchook et al.’s [24] typology, we could not provide a very detailed view of fidelity in terms of content, dose, and coverage.

### A modified Carroll et al.’s framework for implementation fidelity

Therefore, we confronted our empirical research with existing literature on fidelity. As a result, we identified Carroll et al.’s [15] framework for implementation fidelity as the one that could potentially meet our concern of assessing adaptation in the context of fidelity [33].

This framework, slightly modified by adding two moderating factors (i.e., context and participant recruitment), was empirically tested by Hasson et al. [63, 64] in the evaluation of implementation fidelity of a complex intervention in health and social care. Recently, Gagliardi et al. [65] and von Thiele Schwarz et al. [12] applied the conceptual framework to surgical safety checklists and occupational health interventions, respectively. In all the above studies, the authors acknowledged that the framework was a useful evaluation tool for implementation fidelity for complex interventions.

**Fig. 2:**
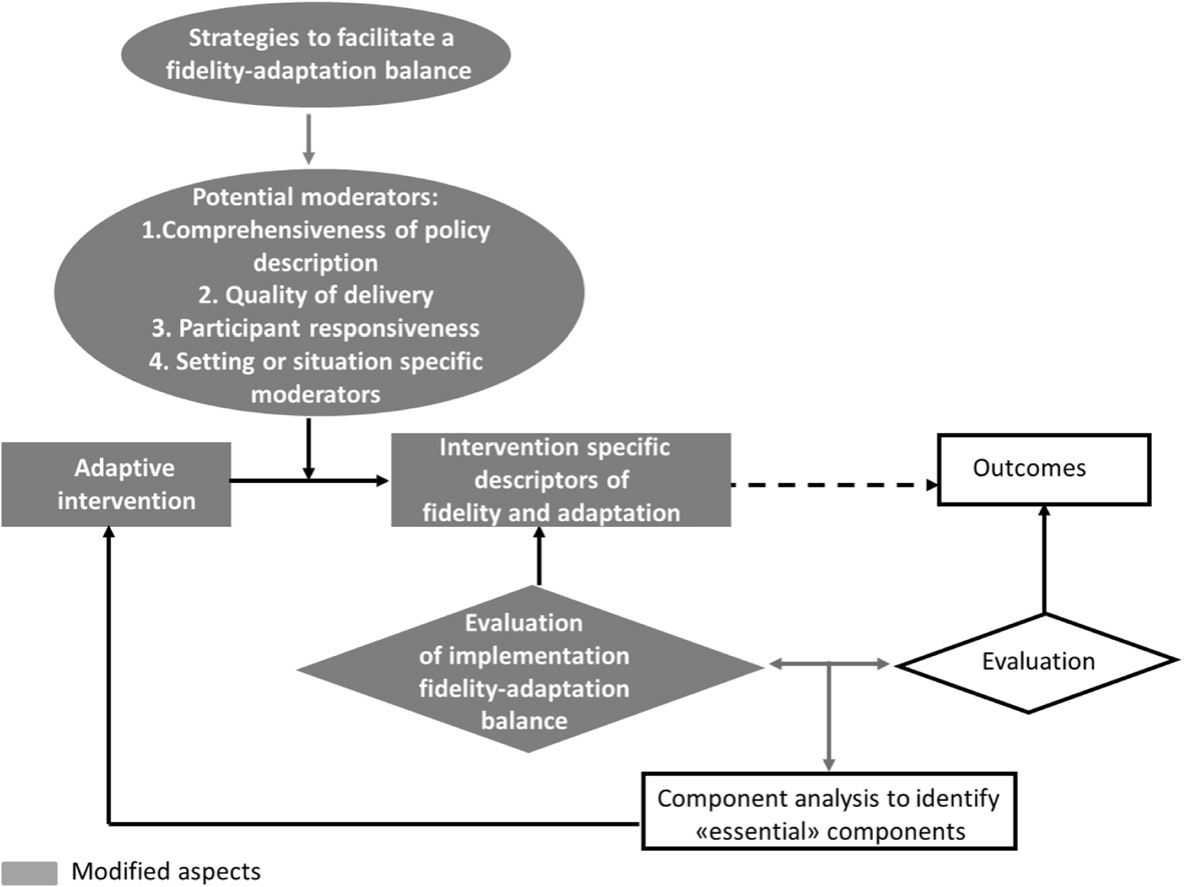
Modified Carroll et al.’s conceptual framework for implementation fidelity

We propose a modified Carroll et al.’s [15] framework for implementation fidelity that retains these authors ideas of conducting outcome evaluation and component analysis to identify those elements that are essential for an intervention. Indeed, evaluating implementation efforts and measuring the fidelity-adaptation balance have a meaning only in the context of outcomes [11]. However, we improved the graphical representation of the role of fidelity and outcome evaluations in identifying the essential components of an intervention (Fig. 2).

We propose some major modifications to the framework. Carroll et al. [15] established adherence as the sole bottom-line measurement of implementation fidelity. As we learned from our empirical fidelity research [61], the nature of adaptations needs to be consciously captured in relation to their effect on effectiveness. In the same line, von Thiele et al. [12] recently suggested applying Carroll et al.’s subcategories of adherence (i.e., prescribed content, frequency, duration, and coverage) to describe and categorize adaptations. In addition, subcategories of adherence might not always be sufficient for every particular intervention; other aspects might be required.

Thus, in our adapted framework, we propose assessing intervention specific descriptors of both fidelity and adaptation, instead of fixed subcategories of adherence alone. To move in this direction, intervention developers need the following: first, to have a clear idea of the expected outcome(s); second, to make the functioning principles or theory of change [42] of the intervention explicit; third, to be able to state them in the form of specific descriptors of fidelity; and fourth, to establish questions to identify adaptations based on the description of the intervention. Intervention specific descriptors of adaptation are developed through answering these questions. A step further is to determine to what extent the adaptations identified affect the functioning principles of a particular component and/or of the intervention as a whole.

An example of this reasoning is provided using the capacity-building component of our empowerment strategy. The expected result was that the participants acquire the knowledge and skills to change the existing power relationships among them. The functioning principles were rooted in the pedagogical model of popular education [66]: the participants of the training need to acquire specific knowledge and skills, these are provided through a participatory learning process (learning group); and the learning group has to involve stakeholders who need to change their power relationships.

In Table 2, we provide specific descriptors of fidelity for capacity building. These provide a comprehensive description of the intervention as intended with details on content, processes (e.g., “what,” “how,” “how frequently,” “to whom,” and “by whom”) and specifications related to the implementation context. In Table 3, we provide questions to identify if adaptations were brought to the capacity-building component. Taking into account, the functioning principles of the component, only the first adaptation identified distracts from the expected result.

**Table 2:** Example of specific descriptors of fidelity for the capacity-building component of the empowerment strategy for dengue vector control. La Lisa municipality, Havana City, 2004–2007

Specific descriptors of fidelity for capacity-building	
What: development of knowledge and skills on four topics: (1) diagnosis, group work, and participation; (2) surveillance of risks and behaviors; (3) action plans and communication strategy; and (4) participatory evaluation.	
How: through a workshop based on the principles of the pedagogical model of popular education: e.g., the objective is that the participants think and, consequently, transform their reality, using a dialectic logic between theory and practice and participatory and experience-based learning methods.	
How frequently: one 4-h workshop for each topic in a 3-month-span period.	
To whom: a learning group composed of three to five stakeholders with different power relationships in relation to dengue vector control activities, belonging to at least three communities.	
By whom: facilitators previously trained based on the principles of the pedagogical model of popular education.	
Specifications related to the context: Three PCs are involved in the project. There are five to six circumscriptions randomly selected per PCs. Methodological support for the training is provided: e.g., written guidelines on how to conduct a popular education workshop, methodological counseling to the facilitators by at least one IPK’s strategy developer.

**Table 3:** Example of specific descriptors of adaptation for the capacity-building component of the empowerment strategy for dengue vector control. La Lisa municipality, Havana City, 2004–2007

Specific descriptors of adaptation for capacity-building	
Questions to identify adaptations	Specific descriptors of adaptation for a PC
What: Was the content of the training changed in any way? How? Was any topic suppressed? Which one? Why? Was any topic replaced? By which one? Why? Was any topic added? Which one? Why?	The topic “diagnosis, group work, and participation” was suppressed of the content of the training because it was deemed irrelevant by the facilitators. Monothematic workshops on communication strategies were added to facilitate the assimilation by the participants of the topic.
How: Was any principle of the pedagogical model adapted (e.g., objectives, logic, learning methods)? Which one? How? Why? Was the pedagogical model replaced by another? By which one? Why?	Some of the learning methods were adapted to the characteristics of the participants. Reading sessions were replaced by interactive lectures to facilitate the understanding of the topics.
How frequently: Was any adaptation introduced in the frequency of the training (e.g., number of sessions, number of hours per sessions? How? Why? Were the workshop’s sessions split over time? How? Why? Was there any adaptation introduced in the length of the span period intended to provide the training? How? Why?	No adaptations (i.e., additions, modifications, deletions) identified
To whom: Was the learning group adapted in any way (e.g., quantity of the participants, role of the stakeholders in relation to dengue vector control activities)? How? Why? Was the learning group replaced by another teaching strategy? By which one? Why?	No adaptations identified
By whom: Was any facilitator not trained? Why? Was any principle of the pedagogical model adapted while training the facilitators? Which one? How? Why? Was the pedagogical model replaced by another? By which one? Why?	No adaptations identified
Specifications related to the context: Was there any change in the number of CPs? Why? Was there any change in the number of circumscriptions involved? Was there any circumscription replaced? How? By which one? Why? Were there modifications brought to the methodological support (e.g., provision of guidelines, content of the guidelines, methodological counseling)? How? Why?	No adaptations identified

Through this analysis of the adaptations brought to an intervention, avenues to prospectively improve implementation can be identified. The added value would be to help intervention developers to identify those non-pre-defined adaptations that could improve the design of the intervention and, thus, effectiveness. Once a positive adaptation is identified, the intervention could go through a new cycle of designing, implementing, and testing. This would require feedback mechanisms.

The need to further identify potential sources of variability in implementation has been highlighted by some authors [67, 68], and Carroll et al. [15] themselves acknowledged that the level of fidelity achieved is influenced by potential moderating factors, which are not necessarily independent. Hasson et al. [63, 64] further emphasized the importance of other mechanisms and factors influencing implementation fidelity while testing Carroll et al.’s framework. Our modified framework maintains comprehensiveness of policy description, quality of delivery, and participant responsiveness as basic potential moderators, but permits to include other setting- or situation-specific moderators.

In addition, we agree with Carroll et al. [15] that facilitation strategies could influence potential moderators of the level of fidelity achieved. For instance, providing manuals and training to implementers could improve the quality of the delivery of an intervention. However, in our modified framework, those strategies are put in place not with the purpose to increase “strict adherence,” but to contribute to achieve an adequate fidelity-adaptation balance. Once adaptations and their positive or negative effects have been identified, facilitation strategies will only address those deemed as inadequate.

In the case of adaptive interventions, this also provides scope for adaptation. The need for such an adequate fidelity-adaptation balance, emphasized by Backer [11] and von Thiele Schwarz et al. [12], is strongly supported by the results of our empirical fidelity research [61] and further research on diffusion of the empowerment strategy for dengue prevention [69]. In practice, aiming at a fidelity-adaptation balance implies interdependency between fidelity and adaptation.

We also propose a further minor modification to Carroll et al.’s framework regarding the use of the term *intervention*. Keeping in mind the controversial top-down versus bottom-up approaches to implementation [11, 33], *intervention* may be a suited term for a top-down approach. Therefore, we consider that the term *adaptive intervention* is more appropriate in our modified theoretical framework of implementation fidelity.

More empirical research is needed to test and validate the modified framework, but we do believe that it may permit a comprehensive assessment of the implementation fidelity-adaptation balance for adaptive interventions.

## Conclusions

Translating into practice evidence-based interventions, deals with unresolved tensions between the need for high implementation fidelity to ensure interventions’ intended effects and bringing changes to the original proposal to fit potential users’ needs. We argue that the issue of fidelity cannot be apprehended independently from the issue of adaptation and that both concepts are intrinsically linked. This paper proposes a conceptual framework of implementation fidelity modified from Carroll et al. [15] suitable to assess the fidelity-adaptation balance for adaptive interventions.

We retain Carroll et al.’s ideas of identifying those elements of an intervention that are essential to maintain its intended effects. However, we propose assessing intervention specific descriptors of both fidelity and adaptation instead of fixed subcategories of adherence alone. We argue that the assessment should capture the nature of the adaptations that occur while implementing adaptive interventions in relation to their effect on effectiveness. Besides, the measurement of adherence stricto sensu may not always be applicable to a particular intervention. We also suggest that developing facilitation strategies could influence moderators of fidelity and do not need to serve the purpose to optimize implementation fidelity, but to achieve an adequate fidelity-adaptation balance.

More empirical research is needed to test and validate the modified framework, but we do believe that it may permit a comprehensive assessment of the implementation fidelity-adaptation balance for adaptive interventions.
