# Erratum to: ‘A modified theoretical framework to assess implementation fidelity of adaptive public health interventions’

**DOI:** 10.1186/s13012-016-0471-x

**Published:** 2016-07-26

**Authors:** Dennis Pérez, Patrick Van der Stuyft, María del Carmen Zabala, Marta Castro, Pierre Lefèvre

**Affiliations:** 1 Epidemiology Division, Tropical Medicine Institute “Pedro Kouri”, Autopista Novia del Mediodía, Km. 6 ½, La Lisa, Marianao 13, PO Box 601, Havana City, Cuba; 2 Department of Public Health, Institute of Tropical Medicine, Nationalestraat 155, 2000 Antwerp, Belgium; 3 Department of Public Health, Ghent University, Ghent, Belgium; 4 Latin-American Faculty of Social Sciences (FLACSO), Habana University, Havana, Cuba

Unfortunately, the original version of this article [1] contained an error. The third author’s name was displayed incorrectly as Maríadel Carmen Zabala. The correct version is María del Carmen Zabala, where María del Carmen are the given names and Zabala is the family name.
